# Psychotherapists’ self-protective attachment strategies

**DOI:** 10.3389/fpsyg.2025.1637760

**Published:** 2025-10-03

**Authors:** Michele Giannotti, Patricia Crittenden, Andrea Landini, Furio Lambruschi

**Affiliations:** ^1^Department of Psychology and Cognitive Sciences, University of Trento, Rovereto, Italy; ^2^Family Relations Institute, Black Mountain, NC, United States; ^3^Family Relations Institute, Reggio Emilia, Italy; ^4^Scuola Bolognese di Psicoterapia Cognitiva, Center for Cognitive Therapy, Forlì, Italy

**Keywords:** attachment, psychotherapists, patients, trauma, unresolved loss, dynamic-maturational model, DMM

## Abstract

Attachment describes how people use relationships to cope with exposure to danger. That function is central to psychotherapy. This study used the Adult Attachment Interview (DMM-AAI) to compare psychotherapists’ attachment strategies to those of patients in psychotherapy and adults drawn from the normative non-patient population. The central variables were attachment strategies (treated as dismissing of relationships Type A1-8, secure/balanced in relationships B1-5, and preoccupied with relationships Type C1-8, plus A/C combinations), psychological trauma and unresolved loss, extremes of arousal, and reorganization toward psychological balance and integration (i.e., conscious change toward B strategies). Differences based on professional training (psychodynamic, cognitive, and family systems) were explored for psychotherapists. The results indicated that non-patients demonstrated the lowest risk attachment strategies (i.e., A1-2, B1-5, and C1-2), whereas patients exhibited the highest risk and most extreme attachment strategies (i.e., A5-8, C5-8, and A5-8/C5-8), and the most psychological trauma, unresolved loss, and extreme arousal. Psychotherapists were not a homogeneous group: approximately 40% showed extreme attachment strategies, whereas the remainder demonstrated low-risk strategies. A higher proportion of psychotherapists (24.6%) showed reorganization toward B strategies than patients (6.8%); this replicates earlier work on British psychotherapy students and patients. Trauma and loss were significantly more frequent in both patients and psychotherapists than in non-patients. No differences were identified based on psychotherapists’ theory training. These findings suggest that more than half of Italian psychotherapists have the potential to establish intersubjectivity with their patients whereas almost half might face problems reaching beyond their personal perspective. Suggestions for improving training and supervision of psychotherapists are offered.

## 1 Introduction

Improving the effectiveness of psychotherapy, currently estimated at 40-50%s (Nemeroff, 2020; Cuijpers et al., 2021; Cuijpers et al., 2024; Zilcha-Mano, 2025), is crucial and might require a better understanding of psychotherapists’ contribution to the therapist-patient relationship. Strikingly, psychotherapist-related characteristics and common factors, such as therapeutic alliance, account for almost half of the variance in psychotherapy outcomes. In contrast, treatment technique, psychotherapists’ theory training and demographics contribute only minimally (Baldwin and Imel, 2013; Hill and Castonguay, 2017; Laska et al., 2014; Wampold and Imel, 2015); the relation of psychotherapists’ attachment to treatment outcomes has not been tested. Despite their relevance in shaping intersubjective processes in clinical settings, strategies for responding to danger have received relatively little attention among psychotherapist-related variables.

There is growing evidence that exposure to danger underlies patients’ psychological dysfunction (Felitti et al., 1998; Kessler et al., 1997; Zarei et al., 2021). The concept of psychotherapists as the ‘wounded profession’ (Jung, 1993) suggests that psychotherapists might be similar to patients in exposure to danger, with some evidence supporting this.

Psychotherapists frequently report exposure to negative family environments (Fussell and Bonney, 1990), including a higher prevalence of adverse childhood experiences (Essletzbichler et al., 2024) and psychological trauma (McBeath, 2019) than the general adult population. A particular concern is psychotherapists’ frequent history of child-adult role reversals (Cruciani et al., 2024) which might affect their role with patients.

We used attachment to address psychotherapists’ protective attachment strategies for coping with danger. Attachment is a multi-faceted variable that addresses response to danger as well as individual differences in current functioning that might affect psychotherapists’ work with patients (Crittenden et al., 2021a,b). Crucially, attachment is an *interpersonal* construct that is relevant to psychotherapists’ functioning with their patients. For everyone, protective attachment strategies can be used to protect the self or an attached person. In psychotherapy, therapists should function as transitional attachment figures, using their strategies to protect their patients until the patients are able to function adaptively and independently. This study compares the protective attachment strategies of psychotherapists, patients, and the normative population, exploring the similarities and differences among these three groups.

### 1.1 Individual differences in attachment

Understanding individual differences in attachment has changed greatly since Bowlby introduced the ideas of *anxiety* regarding uncertain danger, often leading to preoccupation with danger (Bowlby, 1979) and *inhibition* from loss, often leading to dismissing of danger and, sometimes, depression (Bowlby, 1980). Ainsworth’s work with infants (Ainsworth et al., 1978) led to three primary categories of individual differences in attachment: avoidant/dismissing (A), secure (B), ambivalent/preoccupied (C)^1^. Main and Solomon (1986) added a category called ‘disorganization’ (D), for excessive fear resulting from unresolved trauma or loss. In Main’s system, preoccupied (C) and dismissing (A) were risk categories and disorganization (D) was high risk. However, empirically disorganization greatly overlapped with preoccupied and was confused with B (Crittenden et al., 2021a,b). Eventually D was set aside as insufficiently valid by those who had published research using the category (Granqvist et al., 2017).

Concurrently, Crittenden defined a series of subtypes of A, B, and C, leading to a set of five subtypes of secure (B1-5), eight subtypes of dismissing (A1-8), and eight subtypes of preoccupied (C1-8), plus the combination of dismissing-and-preoccupied (Crittenden, 2016); this model was called the Dynamic-Maturational Model of Attachment and Adaptation (DMM). The ABC strategies represent a gradient between reliance on cognitive/logical information and affective arousal to organize behavior. The B strategies balance the use of cognitive logical information or affective arousal, with B3 being perfectly balanced, B1-2 leaning toward A and B4-5 leaning toward C. The A strategies emphasize cognition at the expense of feelings whereas the C strategies emphasize feelings at the expense of logic. The higher the number of the A or C strategy the greater the imbalance of cognition and affect. The DMM considers the attachment categories to be *strategies* for identifying and protecting the self from danger. In the DMM classificatory system, A1-2, B, and C1-2 are low risk categories, A3-6, C3-6, and A3-6/C3-6 are risk categories and A7-8, C7-8, and A7-8/C7-8 are high risk categories.

### 1.2 Assessment of adult attachment

The notion that individual differences in information processing underpinned differences in behavior (Bowlby, 1980) became the basis for the *Adult Attachment Interview* (AAI, George et al., 1985). The interpersonal quality of the AAI is particularly important for studying the therapeutic relationship because the AAI (a) asks about past protective relationships, (b) uses an interpersonal process with an interviewer to elicit the speaker’s enacted protective strategy, and (c) has the potential to suggest the strateg(ies) the speaker might use in other attachment relationships, such as therapeutic relationships. The AAI is a semi-structured interview that queries respondents about their childhood experiences; the questions probe particular aspects of information processing, e.g., semantic generalizations, episodes. The AAI has become the most widely and valid used instrument for assessing attachment in adulthood (Leak and Parsons, 2001).

There are two primary methods for analyzing the AAI: the Berkeley method (Main et al., 1984-2003) and the DMM adaptation of the Berkeley method (Crittenden and Landini, 2011). Both methods rely on discourse analysis to assign individuals to an attachment classification, but differ in the number of outcome classifications and use of rating scales. The Berkeley method has four outcome categories: dismissing (A), secure (B), preoccupied (C), and cannot classify (encompassing disorganization, unresolved trauma and loss, and dismissing/preoccupied combinations). For the Berkeley method, the discourse is coded, then rated on several scales in three memory systems (semantic, episodic, and integrative; Tulving, 1979) that yield a classification. The DMM method has added discourse markers to the Berkeley method and clustered these into six memory systems (procedural, imaged, and connotative being added; Tulving and Schacter, 1990). These yield a *protective strategy*; rating scales are not used. In addition, the DMM classifications can include *interrupters*, i.e., indicators of unresolved loss and psychological trauma; these were denoted when the past danger interrupted current strategic behavior. In some cases, the strategy as a whole is *modified* adversely by pervasive extremes of arousal: ‘depression’ for low arousal and ‘disorientation’ for high arousal. In addition, a strategy can be modified favorably by ‘reorganization’ toward B, that is, the speaker is aware of using a distorted strategy and of consciously changing it. A DMM classification, thus, has three parts: (a) a protective strategy – in all cases, (b) interrupters – or not, and (c) a modifier – or not. Interrupters and modifiers raise the risk associated with the strategy. Notably, every refinement of the 3-category method has reduced the proportion of B classifications, suggesting that ‘false Bs’ were more frequent in earlier classificatory methods.

### 1.3 Psychotherapists’ attachment organization

Only a few studies have formally assessed psychotherapists’ attachment with mixed outcomes regarding their classifications.

Using the Berkeley 3-category classificatory method (reduced to a secure/anxious dichotomy), a German study of 22 psychotherapists classified 64% as secure (Petrowski et al., 2013). Among the anxious group, patients’ attachment to their psychotherapists was found to reflect the psychotherapists’ own avoidance or preoccupation (Petrowski et al., 2013). Similarly, an Italian study of 50 psychodynamic psychotherapists classified 64% as “secure/autonomous,” 24% “dismissing” and 12% “preoccupied” (Talia et al., 2020). Among 31 German psychotherapists assessed using the 4-category Berkeley method, 61.3% were classified as secure, with 22.6% unresolved/disorganized (Schauenburg et al., 2010).

A Brief Report of 11 British psychotherapists-in-training and 15 patients collapsed the DMM subclassifications into three ABC categories (Hughes et al., 2000). The classifications of the primary coder (PMC) showed 36% B, 55% reorganizing from A to B, and 9% A/C for psychotherapists; the patients were distributed as 0% B, 13% reorganizing to B, 40% C, and 47% A/C (Crittenden, 1988). The patients were at all stages of therapy from beginning to closing. Notably, there was no correspondence between AAI classifications and classifications of transcribed therapy sessions (patients) or therapeutic interviews (psychotherapists), probably because the therapy questions primarily probed semantic memory without episodic comparisons (Crittenden, 1988). These data suggest that, although many psychotherapists and patients were reorganizing, more psychotherapists were in the process.

Moreover, based on AAI dimensional ratings derived from the Attachment *Q*-set (Kobak, 1989), an American study of 18 psychotherapists and their 27 clients found higher security in psychotherapists as compared to clients (Dozier et al., 1994). Another American study showed that complementary dismissing versus preoccupied psychotherapist/patient combinations had better outcomes (Tyrrell et al., 1999).

In spite of methodological heterogeneity and weaknesses, it appears that psychotherapists often had insecure attachment and that this might negatively influence psychotherapy (Degnan et al., 2016; Dinger et al., 2009).

### 1.4 Aims and hypotheses

The purpose of this study is to examine psychotherapists’ protective attachment strategies by comparing them with those of patients in psychotherapy and the normative non-patient adult population. We chose to use the DMM classificatory method because of its greater differentiation of individual differences and greater empirical validity, especially in clinical cases (Crittenden et al., 2021a,b).

#### 1.4.1 Hypothesis 1: attachment strategy

Our main hypothesis was that psychotherapists, patients, and non-patients would differ in protective attachment strategies. We expected (H1a) to find a higher proportion of B strategies in the non-patient group and more frequent A/C strategies in the patient group. This analysis was intended to permit a rough comparison with 4-category results reported by others.Clustering the DMM strategies by risk (H1b), we expected the non-patient sample to show the lowest risk and the patient sample the highest (‘high-risk’). Based on clinical experience and the notion of psychotherapy as a ‘wounded profession’, we expected psychotherapists to show a bi-modal distribution, with both well-integrated (Type B) and distressed (high-numbered Types A and C) strategies.

Finally, we computed a quasi-continuous attachment risk variable (H1c) to confirm H1b. We expected a reduction in security from non-patients to psychotherapists, to patients. Significant differences were not expected between psychotherapists and non-patients.

#### 1.4.2 Hypothesis 2: psychological trauma, unresolved loss, and modifiers

We expected that patients would have the highest rates of psychological trauma (H2a) and unresolved loss (H2b), and the non-patients the lowest. Patients would also show more frequent markers of extreme arousal (H2c) compared to the other groups.

#### 1.4.3 Hypothesis 3: reorganization

We expected a higher proportion of psychotherapists to be reorganizing than either patients or non-patients.

#### 1.4.4 Hypothesis 4: psychotherapists’ theoretical orientation

We did not expect psychotherapists’ theoretical backgrounds to be related to their attachment strategies, psychological trauma and extremes of arousal.

## 2 Method

We used a multi-group cross-sectional design comparing the DMM-AAI classifications of Italian psychotherapists, patients in psychotherapy and non-patients.

### 2.1 Participants

Participant data were obtained from the archives of the Family Relations Institute (FRI; Reggio Emilia, Italy). Patients (*n* = 133; 56% female) had clinically significant psychological distress or a formal psychiatric diagnosis (e.g., psychosis, personality disorders, mood or anxiety disorders, or sexual dysfunctions). The psychotherapists (*n* = 61; 60% female) had psychoanalytic (*n* = 20), cognitive-behavioral (*N* = 19), and family systems (*n* = 22) training. Normative, non-patient participants (*n* = 128; 66% female) did not report clinically significant psychological distress or any psychiatric diagnosis. There were no differences in age, but there were missing age data on 58.3% of the sample.

### 2.2 Procedure

Each participant was contacted twice, once to obtain consent and once for the AAI to be administered. The consent signed by the participant was retained by the interviewer who then passed to the research their statement that the participant had given permission for their AAI to be added to the FRI archive. Each statement included basic demographics.

The AAIs of patients and non-patients were delivered by 132 professionals taking the *AAI* course to meet the course requirement of learning to administer an *Adult Attachment Interview*. The *AAI*s of psychotherapists were gathered specifically for this study with informed consent from each psychotherapist. These AAIs of psychotherapists were delivered by 8 interviewers. Their AAIs were added to the FRI archive.

The recordings were transcribed verbatim with the transcriptions being classified by blinded and reliable Italian-speaking coders. The classifications included the speaker’s strategy (A1-8, B1-5, or C1-8, plus A/Cs), any psychological traumas or unresolved losses (12 defined types), or any modifiers (excessively high or low arousal and, separately, reorganization).

The study was conducted in accordance with the standards of the Helsinki Declaration.

### 2.3 Data analysis

The 22 DMM strategies were clustered into four main categories (A, B, C, and A/C) and level of risk (low, moderate, and high). In addition, following prior work in the field (Spieker et al., 2021; Giannotti et al., 2022), a quasi-continuous risk variable was calculated from (1) pattern of attachment (i.e., B3 = 1; B1-2 & B4-5 = 2; A1-2 & C1-2 = 3, A3-4 & C3-4 = 4, and so forth), and (2) the presence of psychological trauma or loss and/or extremes of arousal which increased the risk.

To test hypotheses H1a and H1b, we performed two chi-squared tests to examine potential group differences between psychotherapists, adult patients and normative adults on self-protective strategy both in terms of main classification (H1a) and severity of risk (H1b). We used the residuals method as *post hoc* analyses in order to detect significant cells (Sharpe, 2015). The adjusted standardized residuals were used to determine which cells might be of interest, based on a conservative alpha value of 0.01 (*z* value +/− 2.58). To test H1c, regarding the quasi-continuous attachment risk variable, a non-parametric Kruskal–Wallis test was performed. Dunn tests were used for pairwise comparisons and *ad hoc* results were corrected for multiple comparisons using Bonferroni adjustment.

A series of chi-squared tests were conducted to test group differences related to psychological traumas (H2a), unresolved losses (H2b) and arousal (H2c) and reorganization (H3).

Finally, to explore differences within the group of psychotherapists (H4), we replicated the analyses for each of the dependent variables on strategy, trauma and arousal comparing psychotherapists with different training.

To address missing data on participants’ age, we replicated the analyses using listwise deletion, thereby including only participants with complete data. The pattern of results remained consistent, supporting the validity of the main findings. Data were analyzed using Statistical Package for the Social Sciences; SPSS 25 (IBM Corp., 2017).

## 3 Results

**Figure 1:**
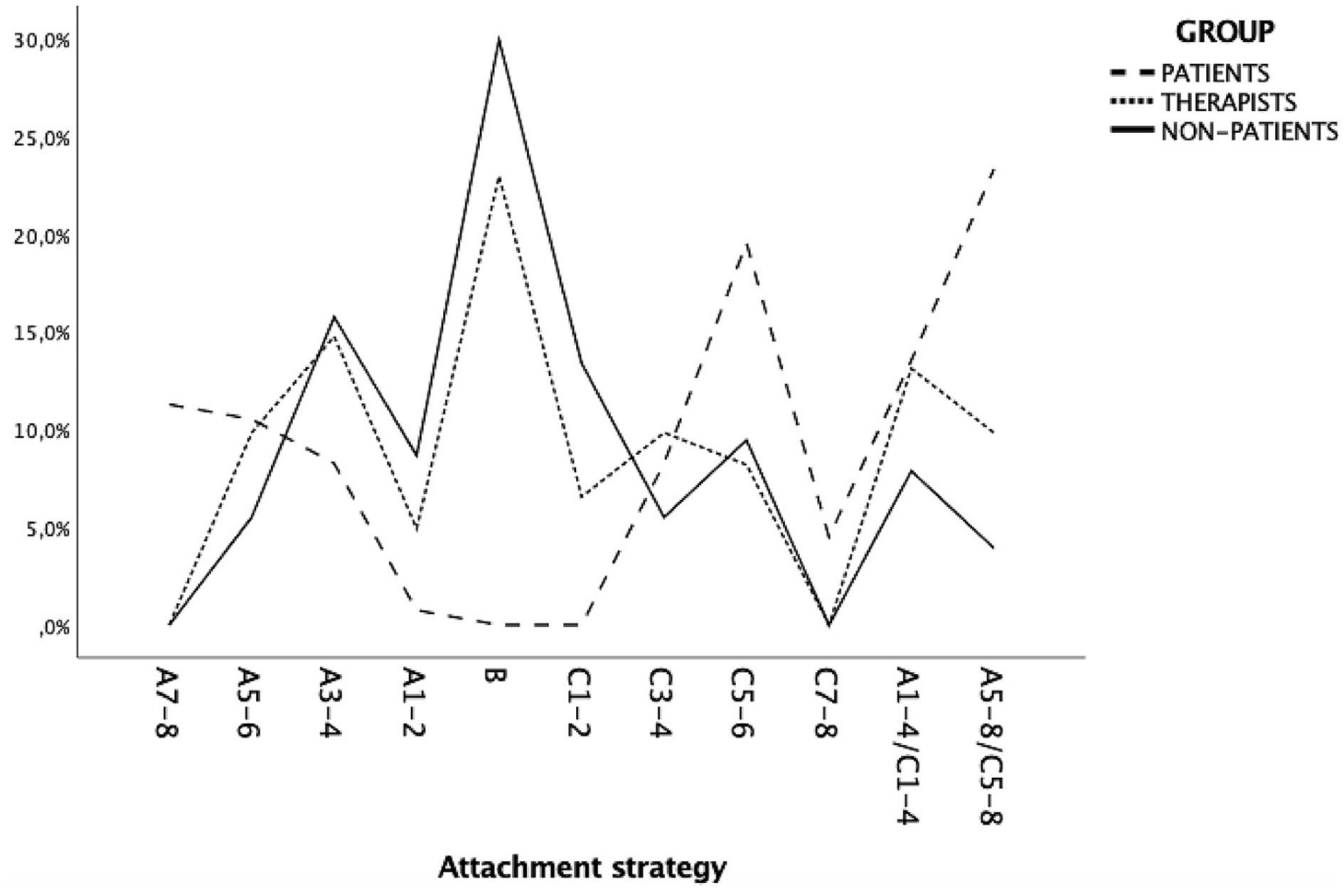
Distribution of DMM attachment strategies among the participant group (*N* = 321). DMM, dynamic maturational model of attachment and adaptation.

The distributions of DMM-AAI attachment strategies among the participant group are shown in Figure 1. Notably, the psychotherapists’ distribution reflected both the low risk of the non-patient distribution and the extreme risk of the patient distribution.

**Figure 2:**
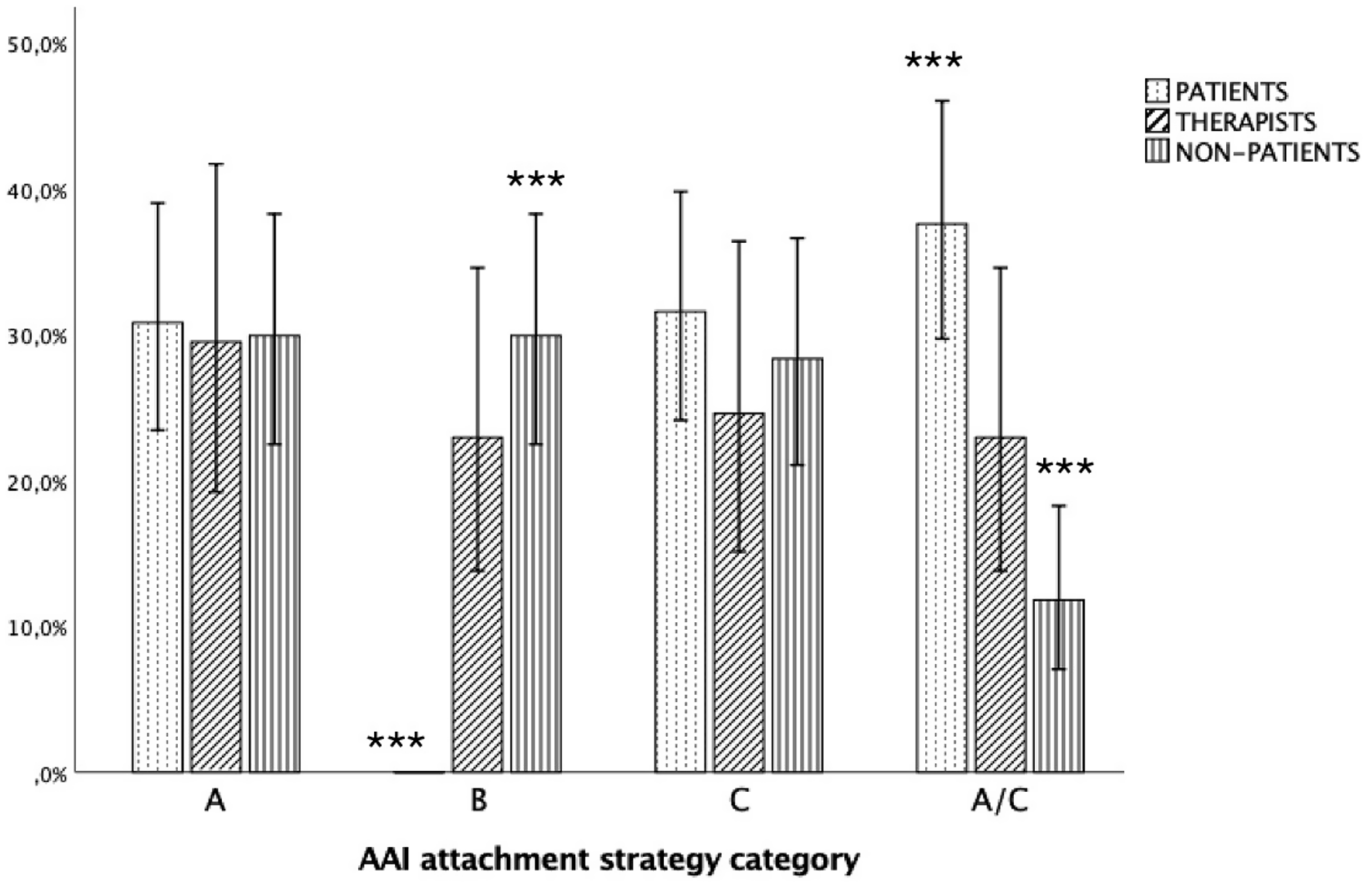
Distribution of A, B, C, and A/C categories across participant group (*N* = 321). AAI, adult attachment interview. ***p < 0.001; 95%, confidence intervals.

Hypothesis 1a (group differences in A, B, C, A/C) was supported [*x*(6) = 56.419, *p* < 0.001, Cramer’s *V* = 0.29] indicating a medium effect (see Figure 2). Non-patients used significantly more B strategies than patients (29.9%, *z* = 5.4, *p* < 0.01), whereas psychotherapists showed a similar trend that did not reach statistical significance (23.3%, *z* = 1.6, *p* > 0.05).

Patients showed significantly higher rates of A/C strategies (37.6%, *z* = 4.5, *p* < 0.01) than non-patients (11.8%, *z* = 4.3, *p* < 0.01), but not than psychotherapists (23%, *z* = −0.3, *p* > 0.05). There were no differences regarding A and C (see Figure 2). Notably, as shown in Figure 1, 14.8% of psychotherapists used an A3–4 attachment strategy, reflecting, respectively, compulsive caregiving or compliance.

**Figure 3:**
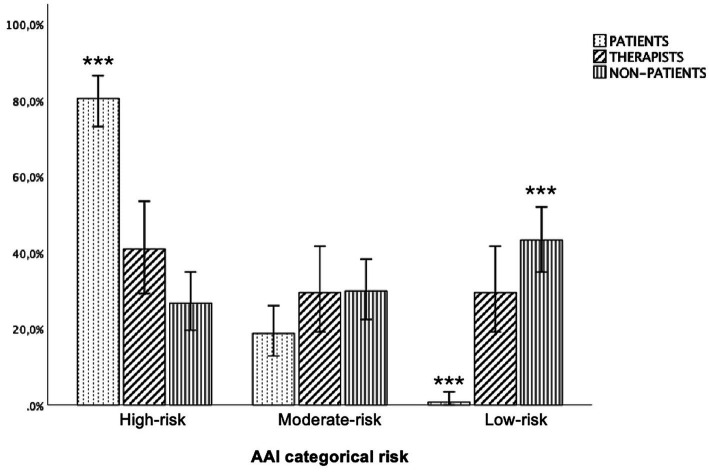
Distribution of attachment strategies by risk category and participant group (*N* = 321). AAI, adult attachment interview. ***p < 0.001; 95%, confidence intervals.

Hypothesis 1b, regarding group differences in risk category, was supported [*x*^2^(4) = 94.00, *p* < 0.001; Cramer’s *V* = 0.38] indicating a medium effect. Patients showed high-risk (*z* = 8.7, *p* > 0.01) and fewer low-risk strategies (*z* = −8.0, *p* > 0.01) than non-patients; patients did not differ from psychotherapists. Only one patient used a low-risk strategy, whereas the majority (80.5%) showed a high-risk strategy. The hypothesis of a bi-modal distribution is only partially supported since psychotherapists mainly showed high-risk (41.0%) and moderate-risk and low risk attachment strategies in the same percentage (29.5% each). Non-patients showed the highest rate of low-risk strategies (43.3%, *z* = 7.0, *p* > 0.01) differing significantly from patients, but not from psychotherapists. There were no significant differences in moderate risk attachment strategies (see Figure 3).

Hypothesis 1c, concerning quasi-linear risk dimension from non-patients through psychotherapists to patients was supported {Kruskal–Wallis test: [*H*(2): 56.49, *p* < 0.001, 𝜂^2^ = 0.171]} representing a large effect. The composite quasi-continuous risk variable showed higher values in patients (*M* = 6.45, SD = 1.91; range = 3–10) compared to therapists (*M* = 4.79, SD = 2.33; range = 1–10) and non-patients (*M* = 3.93, SD = 1.97; range = 1–9). Pairwise comparisons showed that the non-patients (*p* < 0.001) and psychotherapists (*p* < 0.001) had lower attachment risk scores than the patients. Differences were also found between psychotherapists and non-patients (*p* = 0.033), although this result did not remain statistically significant after post-hoc corrections (*p* = 0.088).

Hypotheses 2a and 2b, regarding psychological trauma and loss, were supported. Chi-squared results were significant for psychological trauma [*x*^2^(2) = 76.241, *p* < 0.001, Cramer’s *V* = 0.48], indicating a large effect, and unresolved loss [*x*^2^(2) = 31.740, *p* < 0.001, Cramer’s *V* = 0.31], representing a medium effect. Specifically, one or more psychological traumas were identified in 60.2% of patients (*z* = 8.1, *p* < 0.01), 32.8% of psychotherapists (*z* = 0.3%, *p* > 0.05) and only 8.7% of non-patients (*z* = −7.9, *p* < 0.01). The test comparing type of unresolved trauma (e.g., dismissed or preoccupied) was significant [*x*^2^(4) = 79.59, *p* < 0.001, Cramer’s *V* = 0.35] indicating a medium effect: patients (51.9%; *z* = −7.5, *p* < 0.01) exhibited more preoccupied unresolved trauma than non-patients (7.1%; *z* = −7.1, *p* < 0.01), but not psychotherapists (26.2%; *z* = −0.6, *p* > 0.05). There were no differences in dismissed traumas.

**Figure 4:**
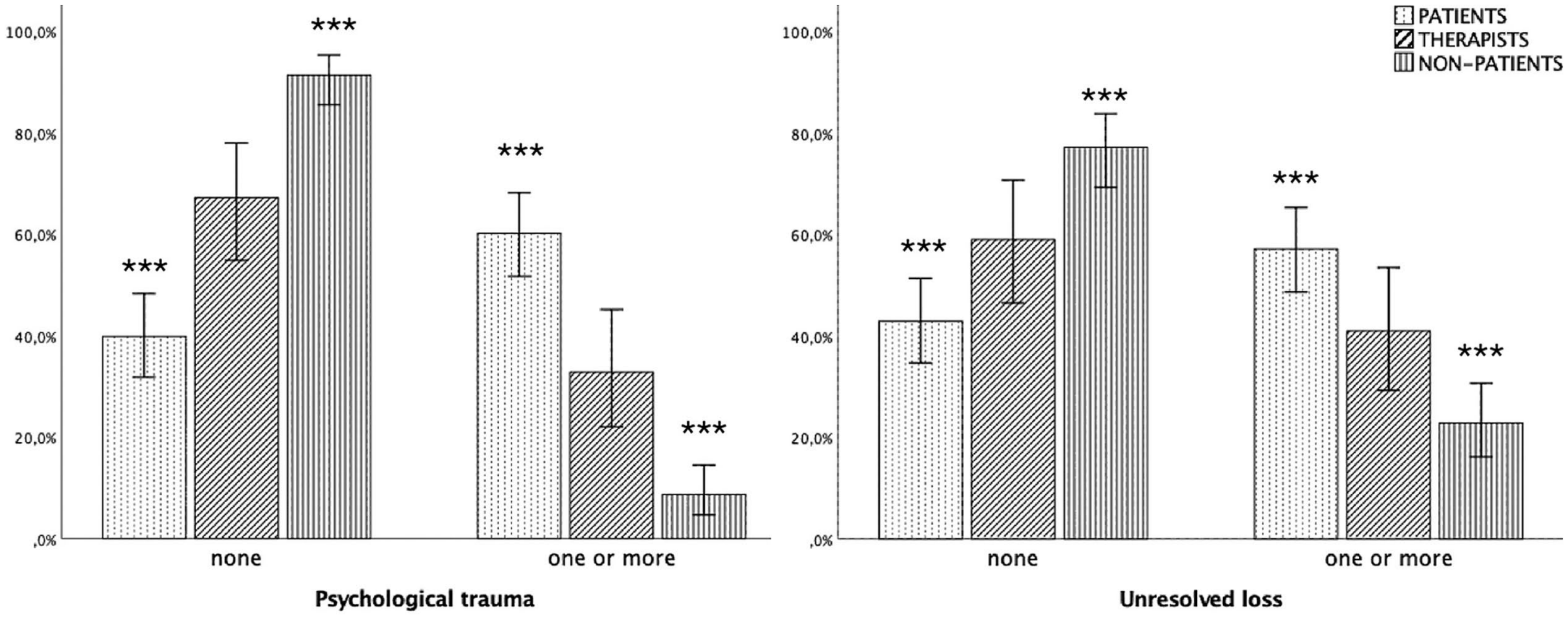
Distribution of psychological trauma (left) and unresolved loss (right) (*N* = 321). ***p < 0.001; 95%, confidence intervals.

Similarly, unresolved loss was more frequent in patients (57.1%; *z* = 5.1, *p* < 0.01) than in non-patients (22.8%; *z* = −5.2, *p* < 0.01). Psychotherapists did not differ from the other groups (41%, *z* = 0.1, *p* > 0.05) (see Figure 4).

Hypothesis 2c, regarding group differences in arousal, was supported [*x*^2^_(6)_ = 46.02, *p* < 0.001, Cramer’s *V* = 0.26] representing a medium effect, with more depression among patients (26.3%; *z* = −5.2, *p* < 0.01) than non-patients (6.3%; *z* = −3.4, *p* < 0.01). Psychotherapists did not differ from either group (6.6%; *z* = −2.0, *p* > 0.05). Non-patients showed less disorientation (high arousal) (0%, *z* = 3.0, *p* < 0.01) compared to both psychotherapists (8.2%; *z* = 1.8, *p* > 0.05) and patients (6.0%; *z* = 1.5, *p* > 0.05).

Hypothesis 3, regarding reorganization among psychotherapists, was supported: a greater proportion of psychotherapists (24.6%; *z* = 3.3, *p* < 0.01) were reorganizing compared to patients (6.8%, *z* = −2.5, *p* < 0.05). There was no difference from non-patients (11.8%; *z* = −0.02, *p* > 0.05). Including reorganization suggests a tri-modal distribution of psychotherapists: those with low-risk strategies (24.6%), those reorganizing toward low risk (also 24.6%), and those using moderate- and high-risk strategies (50.8%).

Hypothesis 4, regarding psychotherapists’ theoretical orientation, did not yield significant differences on any variable (i.e., attachment, risk, unresolved trauma/loss and altered arousal).

## 4 Discussion

### 4.1 The main findings

This study compared protective attachment strategy, interrupters (psychological trauma and unresolved loss), modifiers (pervasive high or low arousal) and reorganization between Italian psychotherapists, patients, and non-patients. The findings showed that non-patients used low-risk protective strategies indicative of greater adaptation, and patients used more extreme strategies, with interrupters and modifiers, indicative of poor adaptation. Psychotherapists reflected three groups. Half used moderate- or high-risk protective attachment strategies, with active psychological traumas, unresolved losses, and depression; this group was similar to patients. A quarter used low-risk strategies. The remaining quarter was reorganizing from high- to low-risk strategies. Our data replicate the unpublished subcategory findings of Hughes et al. (2000) on a British sample 25 years earlier. The higher percentage of B and reorganizing toward B strategies in psychotherapists distinguishes them from patients, possibly reflecting the impact of psychotherapist training and personal therapy on their functioning (Moe and Thimm, 2021) or the therapeutic experience of being a therapist. Finally, there were no differences in the attachment variables for psychotherapists from different theoretical backgrounds.

### 4.2 Questions arising from our unexpected findings

Psychotherapy is a process of promoting adaptative change in patients who have sought such change by engaging in psychotherapy. Other studies indicate that this occurs successfully about 40% of the time (Cuijpers et al., 2021; Cuijpers et al., 2024). Our findings suggest some potential explanations for that low figure.

The most important issue is the impact on their patients of the psychotherapists using moderate- and high-risk strategies. Given the similarity of these psychotherapists’ strategies to patients’ strategies, one could ask whether these psychotherapists offer the benefit of personally informed and helpful compassion for their patients. Alternatively, is it a case of the blind leading the blind? Or, most concerning of all, do some psychotherapists’ strategies protect the therapist from the patient, at the expense of the patient? Of course, all three processes could occur, thus requiring case-by-case analysis. A related question is why there are many more psychotherapists reorganizing their strategies than patients in both our sample and Hughes’ British sample. If psychotherapy is a change process for patients, why did only 7 and 13% of patients show evidence of change while a quarter to a half of psychotherapists showed such change? Who is benefitting from psychotherapy – and how?

Although this is only one study, using a novel assessment, our findings and Hughes’ are consistent with a half century of evidence of the limited effectiveness of psychotherapy (British Psychological Society, Division of Clinical Psychology, 2013; Fonagy et al., 2015). Further, emerging data suggests possible detrimental effects of psychotherapy, with rates ranging from 10–25% (Davidson, 2004; Dimidjian and Hollon, 2010; Lambert and Ogles, 2004; Lilienfeld, 2007; Lohr et al., 2006; Stroebe et al., 2005), but extending as high as 44% in cases of loss and trauma therapy (Fortner, 2000; Kenardy, 2000). Our findings might suggest the processes that result in less therapeutic success than desired. For greater utility, our study should be replicated in different countries with paired psychotherapist-patient AAIs and outcome measures, to account for cultural variations that influence attachment organization.

### 4.3 Implications of these findings for clinical practice

Our findings suggest that therapists’ awareness of their own attachment strategies might be a crucial issue. About half of our psychotherapist sample appears to be strategically organized to avoid such awareness in favor of *self*-protective functioning when therapy becomes threatening for the therapist. Higher self-awareness in psychotherapists might be associated with greater inter-subjectivity (Sidis et al., 2023), reflective integration, and interpersonal sensitivity with patients (Safran and Muran, 2000). Our findings raise three questions: (1) do therapists using moderate- and high-risk strategies have the possibility of delivering effective therapy? and, if so, (2) do their shortcomings outweigh the advantages? Finally, (3) to what extent is psychotherapy training and psychotherapy practice used by psychotherapists to improve their own metal health? Psychotherapists’ active psychological reorganization might in some cases contribute to their therapeutic sensitivity and understanding of clients’ struggles with adverse events. Alternatively, the harmful conflation of psychotherapists’ problems with those of their patients (Norcross and Guy, 2007) might lead to enactments, countertransference burnout, and vicarious traumatization (Newcomb et al., 2015). Future studies could also explore whether psychotherapists’ reorganization makes it easier to relate to patients with less integrated attachment strategies or reflects psychotherapists’ personal benefit from the process of psychotherapy.

### 4.4 Limitations

Comparisons with previous research are challenging due to methodological differences, such as varying assessment tools, coding methods, and cultural background. Missing data on participants’ age constitutes a further limitation of the study. An additional drawback relates to the sample selection methods, because the normative and patient groups were recruited through snowball sampling as part of an AAI training program and the psychotherapists were recruited through psychotherapy training institutes. These methods could introduce potential systematic bias, reducing the generalizability of the results. Future studies should address gaps in the current study by using standardized formal attachment assessments (e.g., AAI) with larger samples of psychotherapists paired with their own patients to examine how psychotherapist and patient characteristics influence treatment processes and outcomes. Other limitations include the lack of data on psychotherapists’ personal therapy, psychotherapists’ experience of adverse events, interviewers’ inexperience with delivering the AAI, patients’ diagnoses, and patients’ stage of treatment, each of which might affect strategy reorganization.

### 4.5 Conclusion and recommendations

This study is unique in several ways. Importantly, we used an assessment tool, the DMM-AAI, that has a wide range of response categories covering the full range of possible adaptation rather than two-, three- or four-category methods that over-estimate security (B). Comparisons with patients and non-patients, using a large sample size, provide stable estimates of group functioning and permit better understanding of psychotherapists’ functioning.

Our finding that psychotherapists’ school of training made no difference in psychotherapists’ protective attachment strategies is consistent with previous evidence that treatment techniques and theory contribute only minimally to treatment outcomes. This suggests changing the way psychotherapists are educated. Because none of the theories has been invalidated, we agree with those who propose integrating the theories’ understanding of etiology and treatment of patients (Castonguay et al., 2015). But integrated approaches, too, seem insufficient to improve the efficacy of treatment. We think a critical idea is missing. Although theories provide frameworks to organize experience, existing models tend to overlook the role of exposure to danger in generating protective attachment strategies and the information processing that underlies them. This leads to several possible changes in training and practice.

Assessment of both incoming students and training curricula should reflect greater emphasis on the effects of danger on information processing. Specifically, the contribution of cognitive-logical and affective information to individuals’ protective strategies should be assessed. Because two-thirds of the experienced psychotherapists in our sample showed a lack of integration around danger and close relationships, this might be of great relevance to trainees. Both group settings and personal psychotherapy could emphasize observing one’s own and others’ protective functioning. The awareness of developmental aspects of brain maturation, both when the danger was experienced and at later ages when psychotherapy is offered or received, could further enhance the psychotherapists’ ability to work with patients and their families.

The high proportion of experienced psychotherapists using moderate- and high-risk protective strategies suggests that increasing psychotherapists’ awareness of their own strategies sufficiently to instigate reorganization toward greater balance might improve treatment efficacy. These psychotherapists, who were likely unchanged by their professional training, might be as limited as their patients in achieving the interpersonal attunement needed for joint problem resolution; supervision could highlight the need for active, experiential practice in interpersonal communication. Further, because these psychotherapists also might find it difficult to identify their patients’ zone of proximal development, thus reducing treatment effectiveness, attention should be directed to signals that patients aren’t engaged. These ‘intersubjective’ skills could reverse some of the negative effects on risk strategies on psychotherapy. Supervision might improve if supervisors were drawn from the low-risk group, especially those who had come from dangerous childhoods and “earned” balanced integration. Finally, self-aware psychotherapists might identify patients or clusters of patients who could benefit from referral to a psychotherapist whose psychological organization meshed more effectively with the patients.

Although disruptive science has dramatically declined (Kozlov, 2022; more than 90% from 1945 to 2010), we recognize that our ideas would mark a major shift in training and practice, constituting a potential bold, even disruptive, contribution. After a half-century of new treatments and theory expansion, possibly it is time for a radical change – beyond theory and new treatment techniques and toward understanding the effects of exposure to danger on both patients and, especially, psychotherapists.

## Data Availability

The datasets presented in this article are not readily available because the data were drawn from the international set of assessment data held by the Family Relations Institute (FRI). Requests to access the datasets should be directed to Patricia M. Crittenden, pmcrittenden@gmail.com.

## Footnotes

^1^Nomenclature is a complex topic in attachment work. Here we use the most basic terminology (secure B, dismissing A, and preoccupied C) because the differences in terminology are not relevant to the findings of the studies.
